# Preservation, modernization, and transformation: contesting bioeconomic imaginations of “manure futures” and trajectories toward a sustainable livestock system

**DOI:** 10.1007/s11625-022-01161-8

**Published:** 2022-06-11

**Authors:** Jonathan Friedrich, Jana Zscheischler, Heiko Faust

**Affiliations:** 1 Leibniz Centre for Agricultural Landscape Research (ZALF), Müncheberg, Germany; 2 Institute of Geography, University of Göttingen, Göttingen, Germany; 3 Department of Geography, Faculty II, University of Vechta, Vechta, Germany

**Keywords:** Imaginaries, Socio-technical transitions, Future visions, Agriculture, Socio-ecological conflicts

## Abstract

In discourses on sustainability, its underlying conceptualizations and meanings, the role of imaginations and their influence on concrete social practices and mutually dependent sociomaterial structures have been overlooked. Therefore, our article uses Adloff and Neckel’s (Sustain Sci 14(4):1015-1025, 2019) conceptual framework to explore the role of imaginations in generating different trajectories from a concrete environmental problem, namely issues attributed to manure surpluses in Germany, to assess the hurdles and conflicting goals of a transformation toward a sustainable livestock system. Our study builds on qualitative, semistructured, and problem-centered interviews with both new innovation actors and incumbent actors in the current system. Our results show that different trajectories of “manure futures” exist, as we identify “preservation”, “modernization” and “transformation” as trajectories representing ideal types of change. We discuss the results in light of the theory of imaginations and reflect on the usefulness of the concept of imaginations for analyzing environmental discourses and practices. Furthermore, we find that normative framings of problems rather than factual knowledge describe contesting imaginations as barriers to sustainability transformations, a point that must be acknowledged when developing a sustainable livestock system. We conclude that contesting imaginations could result in conflicts that must be moderated as drivers for change yet could also point to transformations that are already underway.

## Introduction

The contemporary geological epoch, which is characterized as either the Anthropocene (Lewis and Maslin 2015) or the Capitalocene (Malm and Hornborg 2014; Moore 2017), has increased the pressure on global socioecological systems, thereby creating a need for sustainable change and transformation (Folke et al. 2021; Steffen et al. 2015). However, what exactly this change means remains an open question. Sustainability is a strongly normative concept that has multiple meanings and definitions and can even present conflicting goals (Luks and Siebenhüner 2007; Schneider and Rist 2014; Schneider et al. 2019).

In general, the term “sustainability” is oriented toward the future and thus necessitates imagining (contested) desired future states of social, economic, cultural and ecologic development (Adloff and Neckel 2019; Priebe et al. 2021). Various studies have aimed to cluster the different narratives, imaginations, future visions and potential pathways of sustainability at different scales and spatialities, such as in different sectors or in relation to specific issues, and with different methodologies (e.g., Constance et al. 2018a; Davidson 2014; Knappe et al. 2019; Longhurst and Chilvers 2019; Steffen et al. 2007; Soetebeer 2015). Such attempts include discourses such as those on degrowth, just transitions, green growth, social-ecological transformations and sustainable intensification (e.g., Brand and Wissen 2018; Constance et al. 2018b; Friedrich et al. 2021b; Hickel and Kallis 2020; Kothari et al. 2014; Levidow et al. 2018; Swilling 2020).

Recently, Adloff and Neckel (2019) have introduced a conceptual framework of “futures of sustainability” to grasp how imagined futures, i.e., imaginations, currently influence concrete practices and mutually dependent sociomaterial structures. The novelty of the framework is that it highlights and acknowledges the role of imagined futures in shaping the present in terms of practices and sociomaterial structures. Thus, the framework understands the human embodiment of social practices (following theories of social practices, e.g., Bourdieu 1977; Reckwitz 2002, 2003) as being embedded in imaginations and mutually dependent on sociomaterial structures.

Based on the three analytical categories of social practices, sociomaterial structures and imaginations and their interplay, Adloff and Neckel (2019) conceptualize and differentiate among three different trajectories of sustainability: modernization, transformation, and preservation. The modernization trajectory is shaped by imaginations such as “faith in technological progress”, “adaptation to environmental challenges”, and a “green economy” and thus is manifested in innovation design and results in support for existing socioeconomic structures. The transformation trajectory imagines “real utopias” and a “fundamental societal transformation” and is embodied in practices such as care and sufficiency, thereby aiming to implement new structures to align with the earth system. The control trajectory is based on imaginations such as “technocratic ideals of immunity and resilience”, shaping practices such as geoengineering and surveillance and producing sociomaterial structures of military and state control (Adloff and Neckel 2019). These three trajectories and their imaginations, practices and structures are not mutually exclusive and may intersect in practical arenas of sustainability transformations.

Adloff and Neckel (2019) argue that their theoretical concept must be substantiated with empirical evidence to prove the identified trajectories and the applicability of the sociological concept in specific socioenvironmental contexts. This need is underlined by Delanty (2021), who has called for more empirically grounded research regarding the framework and how imaginations shape “trajectories out of the Anthropocene” (cf. Keck 2021). Furthermore, researching imaginations also allows us to reflect on potential challenges associated with these (desired) future states to modify ongoing change processes. Beckert (2018) has shown that past expectations and imaginations that led to past practices “colonize” the present through, e.g., financial commitments and investments in the economy (see also Friedrich et al. 2022). We argue that, in turn, current practices are not only influenced by future imaginations but can also “colonize” the future, thereby influencing future generations and their living environment. In the context of the sustainability debate, particularly the ethical and philosophical debate, these questions have been described as questions of intergenerational justice (e.g., Meyer 2018).

Against this background, our article uses the German livestock system (see “The case study of the German livestock system”) as a case study of a concrete socioenvironmental issue to prove the empirical applicability of the theoretical concept. This approach allows us to uncover trajectories of “manure futures” and how imaginations shape specific practices and structures of the present and future, thereby contributing to an empirical reflection and substantiation of Adloff and Neckel’s (2019) conceptual framework. In regard to the special issue (cf. Keck 2021), our article contributes to the overarching aim of what futures are imagined and how. Thus, our article answers the following research questions:

- What trajectories of manure futures are visible in the livestock system with respect to imaginations, social practices and mutually dependent sociomaterial structures, and how are they constructed?
- What conclusions can be drawn regarding the design of transformative action and existing barriers for the design of a sustainable livestock system?

First, we will theoretically elaborate on imaginations and their role in explaining and shaping social practices and structures more broadly before presenting the results of our case study. We will discuss our findings in relation to implications for both the theory and practice of researching imaginations and designing a sustainable livestock system.

## Imaginations, social practices and sociomaterial structures

### Clarifications of terms

Research on imagination and imaginaries^1^ has been evoked in recent times based on the work of Castoriadis (1990) on social imaginaries (Adams et al. 2015). Imaginations express fictional future states of living. These include desired states of living and dystopian imaginations that ought not to come into being. However, imaginations not only allow researchers to semantically describe potential future states of living but also arguably influence current social practices and the development of sociomaterial structures (Adloff and Neckel 2019). They can materialize in innovation design as so-called sociotechnical imaginaries^2^ (Jasanoff and Kim 2009; Jasanoff 2015) and are incarnated in economic decisions, ultimately accompanying future expectations (Beckert 2013, 2018).

Imaginations can be described as bundles of hopes, wishes, expectations, and narrations, including moral and affective dimensions (Adloff and Neckel 2019; Beckert 2018). They can be both individually and/or collectively held, but they are socially embedded and constructed. They are fictional in that the future cannot yet be known and is uncertain,therefore, it is described through stories or narratives that ought to happen (Beckert 2018; Esposito 2007). Imaginations are theoretically linked to ideologies. Ideologies, according to Althusser (1968), have an imaginative sphere that captures the relation of subjects to the surrounding material sphere. Here, imaginations are part of ideologies, which are somewhat global and touch upon multiple spheres of subjective and collective behavior.

Beckert (2018) emphasizes that imaginations are oriented toward the future. Nevertheless, they are based on their institutional and social embeddedness, so historical developments are also important in their construction (see also Priebe et al. 2021 for an example of how historical frames of sustainability influence future imaginations of sustainability). Therefore, knowledge and perceived realities such as those described in the problem frames of actors are central to the construction of imaginations at both the individual and collective levels of society. For example, Fladvad and Hasenfratz (2020) show how contemporary and future diagnoses of “unsustainability” mutually interact in imaginations of sustainability. Thus, the imagination of “crisis” leads to other desired futures than, e.g., “normalization”, in that “crisis” specifies the need for change, whereas “normalization” justifies business as usual. Based on these theoretical considerations of imaginations, in the following, we will develop categories from the literature to guide us in answering our research questions.

### Theoretical–conceptual considerations for the analysis of manure futures

**Fig. 1:**
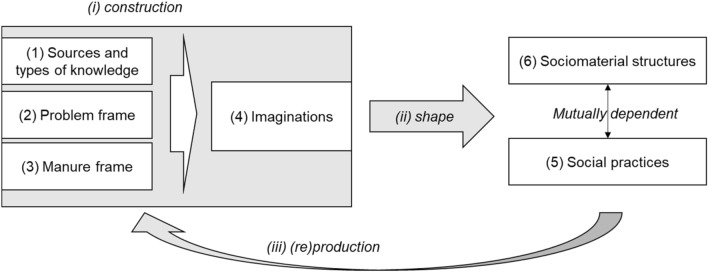
Framework: construction of imaginations and the shaping of social practices and sociomaterial structures

Adloff and Neckel (2019) theoretically elaborate on potential future trajectories of sustainability. As the aim of this study is to empirically uncover different trajectories for the specific socioenvironmental problem of manure surplus (see “The case study of the German livestock system”), we have extended the framework by adding categories from the literature (cf. Adloff and Neckel 2019; Beckert 2018; Berger and Luckmann 1966) to structure our analysis. Adloff and Neckel’s (2019) conceptual framework describes our central considerations, but in an attempt also to grasp the construction, development and embeddedness of imaginations through a social constructivist approach (and to make imaginations empirically tangible), we have extended the framework by understanding imaginations as socially constructed realities^3^ (see Fig. 1, cf. Berger and Luckmann 1966).

As an overview (detailed description below), we understand imaginations (4) as being constructed (i) through sources and types of knowledge (1) as well as the meanings of subjects that are detectable in the frames of problems (2) and manure (3). Imaginations shape (ii) present and future social practices (5) and mutually dependent sociomaterial structures (6), thereby (re)producing (iii) the knowledge and meanings of individuals or collectives (Adloff and Neckel 2019; Beckert 2018; Berger and Luckmann 1966; Longhurst and Chilvers 2019).

Adloff and Neckel (2019) center their theoretical framework around imaginations (4), social practices (5), and sociomaterial structures (6). They argue that imaginations shape and reproduce both practices and mutually dependent structures in society. In their focus on social practices, they build on theories of praxeology as an alternative to theories of action (cf. Reckwitz 2002, 2003). By sociomaterial structures, they refer to infrastructures, such as communication and biophysical infrastructures that are mutually dependent on social practices and (may) need to be transformed to meet imagined futures of sustainability.

As “imaginations tie together cognitive, evaluative, and affective dimensions—knowledge, values and emotions” (Adloff and Neckel 2019, p. 1017), (past) knowledge and experiences are a central category in the construction of imaginations. Following a social constructivist approach, we understand imaginations as imagined (fragmented) future realities, thus, knowledge (1), including the meanings of subjects (2), (3), which is central to the construction of realities, “is the sum total of ‘what everybody knows’ about a social world, an assemblage of maxims, morals, proverbial nuggets of wisdom, values and beliefs, myths, and so forth […]” (Berger and Luckmann 1966, p. 83). Knowledge in this respect circulates within society and is embedded in (sub)worlds of meaning. Accordingly, collectives in society create (fragmented) realities based on their knowledge circulation, the sources of this knowledge (1), and the meanings of subjects (2), (3). Subsequently, individual actors can function as representatives for specific (sub) worlds of meaning, as their knowledge is socially constructed, circulated and embedded 4.

Social practices are shaped by imaginations (Adloff and Neckel 2019), as explained through theories of social practices (also called praxeology), and made observable through interpretive understandings of practical knowledge (Reckwitz 2002, 2003, 2021). For example, technological design as a social practice is shaped by and responsively reproduces imagined realities. Hence, knowledge is also central to explaining the social practices of innovation design (Geels 2020). In this respect, Longhurst and Chilvers (2019) have shown how sociomaterial answers (sociotechnical practice) relate to the contested perceptions of problems (in their framework covered by the “meanings” dimension), which in turn are related to knowledge, values and beliefs because they express the perceived reality of subjects or collectives and are embedded and entangled in sociocultural norms and values (Friedrich et al. 2021a). Problem frame (2) thus functions not only as an example of the meanings of subjects but also as a contemporary diagnosis of the present that marks the starting point for the development of imaginations. Cognitive frames that describe this contemporary diagnosis and build the basis for future imagination are a product of the historical knowledge and experience of both society and individuals (Beckert 2018; Priebe et al. 2021). Thus, practices and structures are not only embedded in imaginations but also reproduce (iii) knowledge and frames of problems (see also Reckwitz 2002, 2003, 2021).

Regarding the context of our case study, by attempting to unravel the trajectories of manure futures, we explicitly integrate the meaning of manure (3), in terms of its cognitive framing, as an individual category that can affect the final imaginations of individuals and collectives. Furthermore, in the design of sociotechnical innovation, the motivation to design an innovation requires assembling the problem frame through the knowledge and experiences as well as the imaginations and expectations of the actors. We thus include motivation as an additional category that refers only to the reasons for developing innovations.

## Methods and research design

This study is part of the research project BioKum (Cumulative effects of bio-economic strategies for a more sustainable agriculture) that aims to gain a better understanding of current sustainability challenges in the German livestock system, with a specific focus on nitrogen surpluses (for a detailed case study description, see “The case study of the German livestock system”). For us, the livestock system, with its practices, complex sociocultural and ecological interactions, diverse perspectives and ethical conflicts, global interdependencies and economic constraints, is a promising unit of investigation for the application of Adloff and Neckel’s (2019) framework.

To uncover the multiple trajectories of manure futures in various regions of Germany, we chose a qualitative approach that followed a deductive–inductive research strategy across twelve problem-centered interviews.

### Data collection

We clustered our interview partner collection according to the multi-level-perspective (MLP) of sociotechnical transitions (Geels and Schot 2007). Thus, our population consists of both (bioeconomic) innovation actors and actors of the existing socio-technical regime (see detailed description below). We followed the working hypothesis that actors in the existing sociotechnical regime^5^ will have different worldviews, knowledge, perceptions of problems, and future imaginations than innovation actors. This fact means that the actors can be viewed as carriers of alternative practices that are embedded in imaginations. As innovation adoption and diffusion depend upon the interactions of niches and the sociotechnical regime (as well as the landscape level), this understanding allowed us to uncover a wide range of different imaginations as well as potential differences and conflicts associated with conflicting future imaginations, practices and structures. Thus, before the interviews were conducted, potential interviewees were clustered based on whether they belonged to the group of bioeconomic innovation actors or actors who constituted the socio-technical regime. Thus, key societal actors included those from policy, science and civil society that constitute the current regime. Bioeconomic innovation actors were defined as people or institutions that had been or currently were designing new practices for reusing or recycling manure, including the development of new products, new processes, substitute products or new behaviors (Bröring et al. 2020). A list of actors creating sustainable manure solutions was based on online research on this topic and included actors from academia and the economy. For both groups, a list of potential interviewees was specified on the basis of online research and discussion. Based on this list, snowball sampling (cf. Reed et al. 2009) was used to identify potential additional interviewees, starting with actors who were randomly chosen beforehand. The interviewed actors are representatives of the organizations we identified through our sampling. There was no spatial focus on one specific region, however, because the manure surplus occurs predominantly in the German states of Lower Saxony and North Rhine-Westphalia, actors from this area were disproportionately included in the sample. Table 1 specifies the interviewed actors.

**Table 1:** Overview of the interviewed actors incorporated into the sample (interviewees are representatives of the respective actors)

Actor description	Number of actors interviewed
Bioeconomic innovation actors from the economy	4 (IP 1, 2, 5, 6)
Bioeconomic innovation actors from science	2 (IP 3, 4)
NGO actors representing nature and environmental conservation	2 (IP 7, 10)
Organic farmers organization	1 (IP 9)
Farming consultancy	1 (IP 8)
Farmers organization	1 (IP 11)
Water suppliers organization	1 (IP 12)

In total, twelve interviews were conducted between May 2020 and October 2020: six with innovation actors and six with actors associated with the socio-technical regime. Due to the COVID-19 pandemic, we avoided personal meetings and conducted online video interviews. The interviews took between 39 and 82 min and were conducted in German 6. The interviews were stopped after content saturation was achieved. This principle applied to recurring arguments that were similar to the content of prior interviews.

During the process of conducting the interviews, we realized that it was impossible to separate the collective and individual opinions of our interviewees. While we aimed to understand the opinions of collectives represented by the corresponding actors, the empirical reality showed that these are ultimately intertwined with personal experiences and perspectives. Thus, both sides are subject to a co-constitutive relationship, and separation is ultimately not possible. We, therefore, understand the presented views of the actors as hybrids of collective and individual opinions that also result from experiences and social entanglements beyond the institutional context.

### Data analysis

**Fig. 2:**
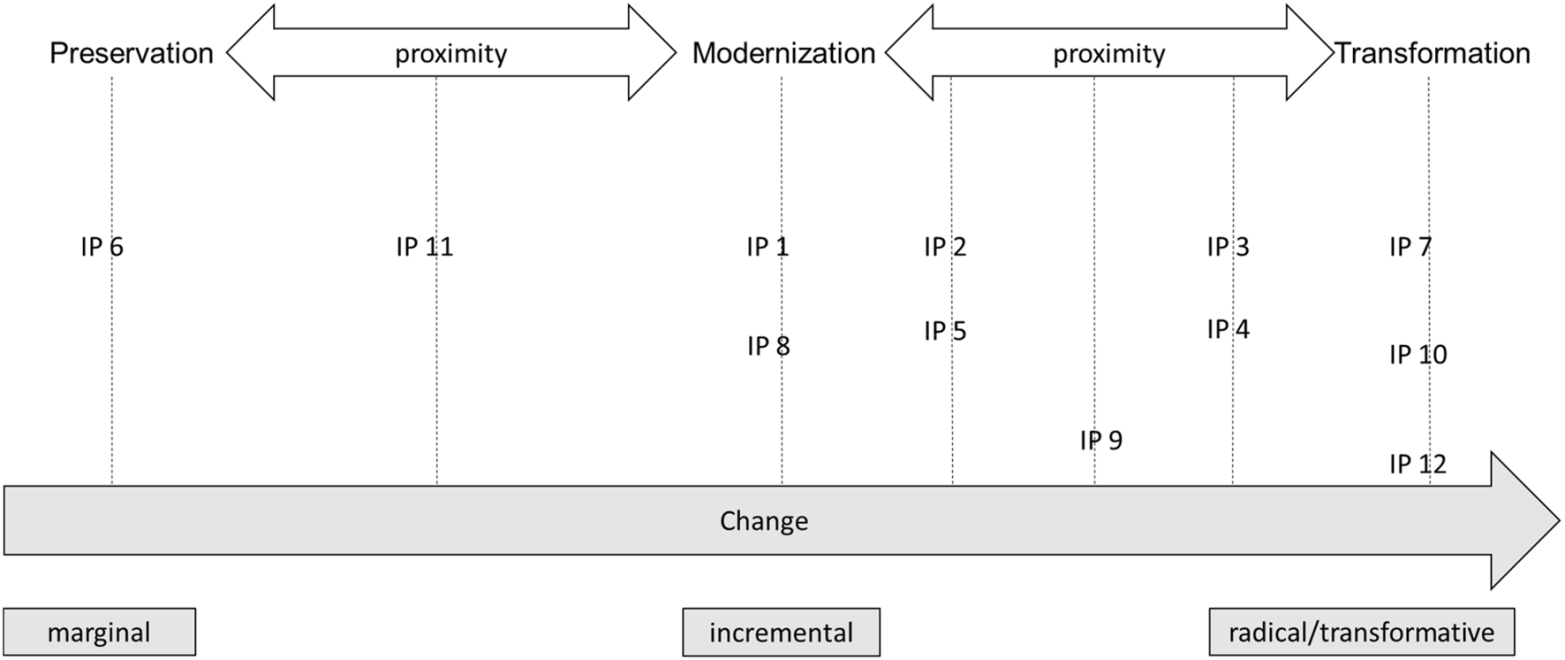
Proximity of interviewees to trajectories (preservation,modernization, transformation) and scope of change

All interviews were recorded, fully transcribed, evaluated, and interpreted according to the “type-building qualitative content analysis” guidance from Kuckartz (2014). Data processing was performed with “MAXQDA” software. The analysis was based on an iterative deductive–inductive research strategy. In the first step, we coded the data deductively by applying the deductive category of the analytical framework developed in Fig. 1. These categories describe the attribute space (Kuckartz 2014). In the second step, additional inductive categories were derived from the material to build subcategories for the attribute space (Fig. 1) in response to the specific empirical example. We followed the proposed trajectories of Adloff and Neckel (2019) in building our types but also refined the category system for the material through coding. Additional trajectories were identified through “polythetic type building” (Kuckartz 2014). The individual cases were attributed to the trajectories in relation to their proximity (see Fig. 2). The coder subjectively located the cases in the graph by qualitatively matching the content of the ideal types (see also Kuckartz 2014).

## Case study and results: contesting manure futures as preservation, modernization, and transformation

The next section first introduces the specifics of our case study in the German livestock system (“The case study of the German livestock system”) before we present our empirical results. As an overview, based on the analysis of the qualitative interviews, we identified three different trajectories (see Table 2): preservation, modernization and transformation. The following sections (“The preservation trajectory”, “The modernization trajectory”, and “The transformation trajectory”) describe the trajectories in detail. As these trajectories present ideal types of change, “Distribution of actors among trajectories” describes the proximity of the interviewed actors to these ideal trajectories.

**Table 2:** Overview of trajectories of “manure futures”: preservation, modernization and transformation; for attribute space description, see Fig. 1 and “Clarifications of terms”

Attribute space	Trajectory		
	The preservation trajectory	The modernization trajectory	The transformation trajectory
(1) Sources and types of knowledge integrated	Reductionist: work context (experiences, networks); scientific sources	Intrasectoral: work context (experience, networks)	Diverse/complex: scientific sources; discussion formats; societal debate; work context
(2) Manure problem frame	Legal rules of application; other actors are responsible; no manure problem (anymore)	Stakeholder interest; deregulation; globalization; nitrogen cycle; legal rules of application	Integrative: usage of manure; environmental issues; nitrogen cycle and planetary boundaries; deregulation; globalization; social consumption
(3) Manure frame	Resource; fertilizer	Recyclable material; resource; fertilizer	Resource; fertilizer; “environmental disaster”
(4) Imaginations	(Economic) growth; preservation of status quo	Green growth; technological fix/faith in technological progress; sustainability through spatial decoupling and closing the loop	Fundamental transformation; changed human-nature relationship; challenging economic growth; (dystopia)
(5) Practices	Free market; innovations: transport and recycling; politics that are reliable for farmers	Innovations: recycling, circularity; free market; labels of sustainability; political support for innovations; science-based practices	Innovations: circular, recycling; consumption practices (sufficiency); cultural change; regulations/laws
(6) Structures	Preservation of existing structures	Adaptation of existing structures; structural support for innovations	Structural change to preserve the value of nature; small-scale agriculture
Motivation of innovation actors	Legal reasons: to meet legal requirements through innovations	Economic reasons: capital accumulation through innovations	Ecological reasons: innovations can contribute to more sustainable agriculture

## The case study of the German livestock system

Most sustainability challenges can be viewed as wicked problems or complex socioenvironmental issues, including multiple practices and politics of unsustainability, which thereby threaten the biophysical conditions of life on earth. These conditions and their limits are well described by the planetary boundaries concept (see Rockström et al. 2009; Steffen et al. 2015).

The livestock system is one in which different dimensions of unsustainability (such as biophysical conditions of life, practices, structures, and politics) become explicit and place pressure on the system to change. There is an ongoing discussion about how to transform agrifood systems, and specifically livestock systems, in (sustainability) science and the public debate (e.g., Franz et al. 2018; Friedrich et al. 2021a, 2021b, 2022; Nowack and Hoffmann 2020; Tamásy 2013). This system is, therefore, a prospective unit of investigation, as it intersects biophysical, social, cultural and societal aspects of sustainability on a local, national and even global level (for a detailed description of the intersections, see below). On the one hand, these entanglements (such as those described in Nexus approaches, e.g., Franz et al. 2018) make it difficult to research, but on the other hand, they offer the possibility of generating results that can be transferred to other socioenvironmental contexts (under consideration of the specific context and system boundaries).

In the following, we outline the current sustainability issues attributed to the livestock system that make it a complex socioenvironmental issue. Biophysical issues include high emissions of methane and nitrous oxides, which contribute to climate change (Tilman and Clark 2014) and fine dust pollution, and nitrate surpluses, which pollute waters and soils, leading to eutrophication. In addition, social issues are attributed to precarious working conditions in the meat processing industry (Friedrich et al. 2021b; Wagner and Hassel 2016), and poor animal welfare has raised questions about ethical responsibility, as have telecoupled effects such as that of land-use change for fodder production in South America on indigenous land rights (Franz et al. 2018; Friedrich et al. 2021a; Sauer 2018). Cultural aspects of diets, such as those associated with meat consumption among individuals or collectives as well as vegetarian or vegan diets, complete the picture of a complex socioenvironmental issue.

To disentangle such complex global interlinkages, we focused our case study on local developments in relation to the nitrogen surplus associated with manure as a product of the German livestock system. Manure and nutrient surpluses are the most perceptible symptoms of an unsustainable system: the odor is well known to neighbors; the eutrophication of groundwater threatens drinking water quality, leading to increased denitrification costs; and the eutrophication of surface water bodies impacts aquatic ecosystems, leads to biodiversity loss (Umweltbundesamt 2019), and even impacts the use of water for bathing. These local issues associated with manure are substantiated by the nitrogen cycle, which has been specified as a high-risk biochemical flow in the planetary boundaries concept (Rockström et al. 2009; Steffen et al. 2015). In addition, legal actions against member states such as Germany by the European Union (EU) have reinforced the pressure to change.

In terms of sustainability-oriented transformations in agrifood systems and beyond, the concept of a sustainable (circular) bioeconomy has recently been evoked (Giampietro 2019). Several policy actors (e.g., the EU and Germany) have published bioeconomic strategies highlighting the role of innovation actors in the development of bioeconomic innovations and international competition in moving toward a bioeconomic future. This role also applies to the case of manure, as bioeconomic innovations such as recycling fertilizer (e.g., Pintucci et al. 2017) and cultivating insects (e.g., Čičková et al. 2015) and duckweed (e.g., Stadtlander et al. 2019) are currently being developed to (partially) close nitrogen cycles with the aim of contributing to a more sustainable agrifood system (Friedrich et al. 2021a, 2022).

In the context of these developments, how manure futures are imagined by these actors, which imaginations guide which practices and the development of which structures, what other ideas from civil society and farmers exist to solve the issues associated with manure and what future these are aiming toward remain open questions.

### The preservation trajectory

The preservation trajectory is characterized by imaginations of a preservation of the status quo; thus, it focuses on economic productivity and growth in the agricultural sector and livestock system (see Table 2). Practices that are shaped by these imaginations are related to regulation aversion in terms of demanding a free market: “The economy always has to come up with something on its own somewhere […]. But if subsidies play a role, if someone somehow applies for something from Brussels or from another side, then that is actually always short-lived. Or even if the state intervenes with restrictions or subsidies, [or] somehow wants to promote something, then that is always only seen quite shortsightedly” (IP 6). However, innovations such as the reciprocal transport of manure and fodder between arable and livestock regions or the recycling of manure are viewed as complementing the existing system and are practices that are embedded in this trajectory. This trajectory also relates to the mutually dependent preservation of existing structures of livestock production and agriculture against the background of feeding the population in Germany (in terms of an obligation): “We believe that Germany, with its favorable production locations, also has a responsibility to use the production opportunities here. And to do so as productively as possible, but also as sustainably and efficiently as possible. So we shouldn’t give ourselves a slender foot in Germany and say we’re going to extensify our production in Germany. And then we import all our food. Instead, we have to use Germany as a production location to ensure security of supply” (IP 11). What is regarded as a manure issue in this regard is the regulation of manure application (such as through the nitrate directive), as manure problems are no longer perceived, as described by IP 6: “And therefore, these surpluses are actually only marginal. So, with these surpluses, to transport them or to get rid of them here, we can always cope with that or have actually already coped with it” (IP 6). The issue of nitrate surplus is thus regarded as a matter of the past. Nonetheless, other sources than livestock farmers are regarded as responsible for the (past) issues that led to the introduction of the nitrate directive—in particular, biogas plants. Manure is framed as a resource and fertilizer as a valuable component of agricultural production. Knowledge of this trajectory is generated in work contexts, such as personal and work-based experiences, and through agricultural networks as well as scientific sources, leading to a rather “reductionist” framing.

### The modernization trajectory

The modernization trajectory is characterized by imaginations of green growth, a technological fix for environmental issues, as is attributed to the manure surplus, and a general faith in technological progress (see Table 2). The green growth imagination relates to the development of innovations that are expected to be highly profitable due to the pressure for solutions that accompanies the manure topic and thus also acknowledges the associated environmental issues: “And I can say that if you have a solution today that works, you’re a millionaire. Because the pressure is just there. So the pressure is immense, and there is no solution that actually helps here” (IP 1). Sustainability is imagined through a spatial decoupling of the production and application of manure and the circular idea of closing loops that both feed into the green growth imagination. Spatial decoupling thereby relates to the introduction of manure-based bioeconomic innovations, such as a circular orientation and manure recycling, which in turn allow manure to be spatially decoupled as a product of livestock production from the legal rules of area-bound application on the field, thereby allowing farmers to close cycles: “That means that we have a surplus of manure here, which is caused by feeding that does not come from here. The cycle is no longer right. That’s why I said earlier: 150 years ago, we had exactly this cycle, didn’t we? […]. And with our technology [recycling fertilizer], you can say that we can do that [close the cycle as we did 150 years ago] on a larger scale. Across farms” (IP 5). As innovations are viewed as pivotal for closing the loops, political support that simplifies the development and bureaucracy that is attributed to processes of innovation development are embedded as practices in these imaginations. Beyond a focus on manure-based bioeconomic innovations and diffusion that relate to this trajectory, further practices are introduced under sustainability labels that accompany consumption and, in relation to manure, document the sustainability of livestock products in regard to the environmental issues hitherto associated with manure usage. In general, the highlighted practices are labeled science-based. These practices relate to the adaptability of existing structures. Such adaptation means adjustments of the sociomaterial structures of production, such as those driven by the bioeconomic, circular innovations of recycling, which change the sociomaterial architecture of manure application and usage without questioning the general model of livestock production. The manure problem is framed as the surplus of manure originating from general economic developments in recent years, such as globalization and deregulation, that have led to open nutrient cycles. Thus, the problem to be solved is specified by the applicable legal rules (in particular the nitrate directive) that must be followed and the broad interests of multiple stakeholders, such as farmers or the mineral water supply: “But the thing that we also see is, with many of them, especially those that have some connection with livestock, and sometimes it’s not so obvious, so also mineral wells, are interested in such solutions. […] So it is now not only necessarily meat or milk production but also there. So there is a broad interest and a broad rethinking to include these things [new forms of production and manure recycling]” (IP 1). In this trajectory, manure is framed as a resource, fertilizer, and recyclable material that has great potential for further use and capital accumulation. The knowledge for these framings is generated in work contexts, namely the experience of the actors and their networks, such as in the search for new development options for one’s own company, which can be characterized as intrasectoral.

### The transformation trajectory

The transformation trajectory is characterized by the imaginations of a fundamental transformation, a changed human-nature relationship, and challenging of the economic growth paradigm (see Table 2). The practices that are shaped by these imaginations are diverse; they include innovations such as recycling and circular-oriented practices (also organic agriculture) as well as practices that can be attributed to sufficiency, such as changed social consumption practices, cultural changes in values and norms, and political rules and laws that aim to conserve the instrumental and intrinsic values of nature. Cultural change is, for example, attributed to consumers valuing sustainably produced goods: “I think the wish for society is that society basically appreciates the production of sustainable food. That society sees when a farmer now contributes significantly more with respect to public things, i.e., water, promotes clean water, and promotes insects or the like. That society is willing to honor that” (IP 10). These practices thus specify a structural change in agricultural production and consumption to conserve the value of nature (intrinsic and instrumental) against the background of visions such as that of small-scale agriculture: “So in any case, already still small-scale agriculture. […] So, small structures make a living possible [for farmers] and just a slow, but still clearly visible, transformation process to fewer animals, more crop rotations, more diversity in the field, fewer pesticides. […] So in itself, we simply need to look at the whole thing again and take out some big adjusting screws and with clear changes in laws, clearly come closer to nature” (IP 7). The manure problem frame is integrative and connects different problems, i.e., the current use of manure, environmental issues such as eutrophication, the nitrogen cycle with respect to planetary boundaries, deregulation, the more general globalization of agricultural production, and the societal consumption of livestock products such as meat: “There’s the flaw in the system. We eat too much meat. We want cheap meat. […] But factory farming naturally leads to this huge amount of manure. And then we really have a problem. It then becomes waste. Or it is then treated like waste, yes. You simply don’t know where to put it” (IP 12). Manure is regarded as a resource and a fertilizer if used correctly, as it can otherwise become an “environmental disaster” (IP 7) or the aforementioned waste (IP 12). The knowledge of this trajectory is diverse and can be characterized as complex, as it is generated by different sources and in different contexts, such as scientific sources, discussion formats with different actors, and societal debate and (interdisciplinary) work contexts.

### Distribution of actors among trajectories

The trajectories of preservation, modernization, and transformation represent ideal types of change that ought to happen. In Fig. 2, the trajectories are organized in terms of how they change the status quo. The interviewees are attached to this grouping in relation to their individual proximity to the trajectories developed above.

With respect to proximity, the background and/or motivation of the interviewed actors is relevant to explaining their position. IP 6, who had already developed an innovation that allowed for the reciprocal transport of manure and fodder between different regions of Germany, argued that this innovation had already led to the solution of the issue; thus, the actor intended to maintain the status quo, as legal requirements were being met, and changes would jeopardize the actor’s business model (see Table 2). In contrast, IP 7, IP 10, the NGO actors, and IP 12, representing water suppliers (not all actors were directly related to agricultural production), argued for a changed human-nature relationship oriented toward conserving both the instrumental and intrinsic value of nature. These actors highlighted the importance of adjusting every practice and structure to align with this goal and thus also called for transforming and challenging a focus on economic growth; thus, all these actors are attributed to the transformation trajectory. The actors IP 1, IP 2 and IP 5 (innovation actors from the economy), who were all close to the ideal type of modernization, were motivated by the potential for capital accumulation through innovation diffusion (see Table 2) while at the same time closing nutrient loops; thus, they are examples of so-called green growth imaginations. IP 3 and IP 4, who were innovation actors from scientific fields, could be called hybrids of the modernization and transformation trajectories. Both were motivated to design innovations for ecological reasons (see Table 2). They called for practices of efficiency and sufficiency, but in contrast to the transformation pathway, they did not highlight the importance of regulations and structural change to align with the values of nature. A similar proximity to both the modernization and transformation trajectories is attributed to IP 9, a representative of an organic farming organization. This actor viewed organic farming as a prototype of how nutrients in the form of manure can be used efficiently to benefit both humans and nature. Although this actor specified the need for change, such as in terms of reduced livestock production intensity, the difference rested in the clear focus on organic farming in contrast to the multiple practices of the transformation trajectory. IP 8, who belonged to a farming consultancy, was proximate to the modernization trajectory, as this actor focused his argumentation on the profitability of farming while at the same time viewing innovations as important to enable farming to adjust to environmental issues such as those driven by the manure surplus. IP 11, who represented a farming organization, was a hybrid of the modernization and preservation trajectories. In contrast to IP 6, who represented the preservation trajectory, this actor viewed the current manure surplus as a regional issue and saw potential in using new innovations. However, this emphasis on the preservation of the productivity of agricultural livestock farming separated this actor from the modernization trajectory, as this emphasis implies only marginal change.

## Discussion

The aim of this article was to identify trajectories of “manure futures” in relation to differing imaginations, social practices and sociomaterial structures and their underlying construction, thereby proving the applicability of the sociological conceptual framework of Adloff and Neckel (2019) to a concrete environmental issue. We identified three different trajectories, namely preservation, modernization and transformation, which are shaped by different imaginations of manure futures, leading to different practices and mutually dependent structures. In the following, we discuss our findings in relation to both reflections and implications for theory, and practical implications for the design of a sustainable livestock system, before reflecting on our methodology.

### Theoretical reflections and implications for the concept of imaginations

Applying the concept of Adloff and Neckel (2019) to the specific case of manure allowed us to cluster different ideas of how to solve the manure issue in terms of trajectories around the concept of imaginations, practices and structures. However, the interviewed actors (see Fig. 2) clearly showed hybrid versions of the trajectories, meaning that these actors embody intersecting trajectories and that a clear, empirical mapping of each actor to one trajectory is not always possible. Adloff and Neckel (2019) have similarly argued that their trajectories could intersect in practical arenas of sustainability discourses. Nevertheless, the identified trajectories and the associated imaginations, practices and structures allow us to conceptually frame the manure discourse and to show which contesting imaginations exist with regard to the design of a sustainable livestock system (see “Implications for the practice and transformations of the current livestock system”).

The results indicate that the approach of Adloff and Neckel (2019) is applicable to specific environmental issues, discourses and actions, such as those found in the German livestock system. When comparing our results with the trajectories of Adloff and Neckel (2019), we identified an additional trajectory: preservation. While the authors (ibid.) start from the hypothesis that society is currently characterized by multiple unsustainable practices, a preservation trajectory that specifies imaginations of no change or only marginal change and builds on neglecting the problem of “unsustainability” does not fit this model. We argue that, on the one hand, a preservation trajectory could be a specific case for the agrifood system, as agricultural sectors are described as highly stabilized through political interventions such as subventions and regulations (e.g., Common Agricultural Policy) to secure productivity. Therefore, actors base their expectations and imaginations on relying on these stabilized architectures, as Barnes et al. (2016) found in studying the livestock system. This architecture is complemented by long-term political-economic path dependencies^7^ that apply to specific regions and sectors and influence the likelihood and scope of imagined change (e.g., Benoit and Patsias 2017) as well as by incumbent actors in the socio-technical regime who reproduce rather than transform current practices and structures of the capitalist system (e.g., Friedrich et al. 2021a; van Oers et al. 2021). In addition, specific actors, such as the German “Bauernverband” (farmers’ association), exercise hegemonic discourse and cultural power in the German agrifood system (see Heyen and Wolff 2019). The farmers’ association has been lobbying ever since its formation to preserve existing hierarchies and resource distributions in the agrifood system, thereby limiting the possibility of sustainable change. This development aligns with what Reckwitz calls the “order”^8^ of social practices that gains a hegemonic character (cf. Reckwitz 2021, p. 72ff). According to Reckwitz (ibid.), it is highly characteristic of modernity that these orders exist in the first place and are being challenged and changed over time. We argue that this fact is also visible in our case study, in which the current prevailing order—namely the preservation trajectory—is being challenged by the emergence of different orders of social practices, as is evident in the transformation and modernization trajectories. This situation could mean that we are currently witnessing a process of consistency building and “undoing orders” (cf. Reckwitz 2021) in the German agrifood system. On the other hand, German society is characterized by multiple practices and structures that are not perceived as “unsustainable” by every individual. Thus, several actors neglect problems such as those associated with the denial of climate change (e.g., Walter et al. 2018) and other environmental issues. We argue that this issue may also apply to the case of manure and the preservation trajectory.

In contrast to Adloff and Neckel (2019), we did not identify a control trajectory. We attribute this difference to the specific case of agriculture. Imaginations that are related to the control trajectory specify far-reaching changes in the whole of society and the elements that constitute it. We argue that this trajectory does not appear in our empirical case due to the scope of changes at the level of every societal organization and constitution that it requires. As Germany relies on federal negotiation processes, another possible reason is that some subsystems are excluded from specific discourses. In our view, further empirical research covering other topics in the agrifood system in Germany could reveal this trajectory (see also “Methodological reflections”).

Due to the empirical context of our case, we extended the framework of Adloff and Neckel (2019) by adding categories. Based on these considerations and with respect to our empirical data, we identified the framing of problems and the framing of manure as central points for explaining different trajectories, their associated imaginations and how these imaginations shape practices and structures. This approach aligns with Delanty’s (2021) critique of the framework of Adloff and Neckel (2019). Delanty (2021) argues that the “relation between actuality, that which exists, and potentiality, needs to be given greater prominence” (ibid., p.7) in the framework. We argue that the problem frame (partially) bridges this gap, as it describes the differing conceptualizations of what is perceived and regarded as the actual problem requiring change. This approach also means that it is not the factual knowledge that constructs imaginations but rather the attributions of meanings by actors, the “normative framings of issues and problematisations” (cf. Longhurst and Chilvers 2019, p. 975). In this context, Reckwitz (2002, 2003) argues that “practical knowledge” is the basis for social practices^9^ in the theory of praxeology. The author (ibid.) conceptualizes “practical knowledge” as knowledge in terms of interpretive understandings. This approach aligns with our theoretical conceptualizations and empirical findings in that the meanings attributed to actors are crucial for the construction of imaginations. We attribute these meanings to the interpretive understandings of Reckwitz (2002). Our study shows that when an actor does not perceive a problem (see the preservation trajectory), the ability to imagine something different is limited. In contrast, viewing the surplus of manure as just one aspect of unsustainability in the agrifood system means having an integrative/complex problem frame—and thus also imaginations that more fundamentally challenge contemporary practices and structures (see transformation trajectory). The framing of manure aligns with that frame, especially in the context of the routine of practices (cf. Reckwitz 2002, 2003) becoming visible, e.g., if manure has always been treated as a fertilizer, this routine also becomes visible in future imaginations and accordant practices.

Another aspect that is visible in our empirical results and that we briefly touch upon in the following (see “Implications for the practice and transformations of the current livestock system”) concerns temporal framings. Some interviewees do not directly relate to imagined futures such that they imagine a reality that is much different from the present. Rather, they rest on experiences and are oriented toward an existing or imagined past. Although interviewees were asked to think about the future, some are more oriented toward the past. This orientation still fits into Adloff and Neckel's (2019) framework, as imaginations can also be oriented toward what has existed and could recover. Thus, imaginations do not necessarily have to adopt utopian or dystopian ideas of what ought or ought not to be; in the end, whatever people imagine regarding the future shapes the social practices they embody. In addition, Reckwitz (2021) argue that late modernity is characterized by temporal hybridization in that societies and their imaginations orient toward different temporal framings, just as different pasts are always accessible through stories, movies, or other historical documents.

### Implications for the practice and transformations of the current livestock system

Priebe et al. (2021) argue that it is not factual knowledge that is missing in designing sustainability-oriented transformations in general but rather that past frames limit societal change such that society is trapped and unable “to examine and challenge prevailing values, habits, and ways of thinking” (p. 82). From a psychological perspective, these tendencies can be attributed to aspects of “system justification” on the individual level (e.g., Feygina et al. 2010). These tendencies are also visible in our case study (see also “Theoretical reflections and implications for the concept of imaginations”). The three identified trajectories and their imaginations, practices and structures do not show new and different ideas of what has been discussed in the public debates of past years. Rather, they present already-existing ideas, such as a fundamental transformation that is often attributed to NGOs or actors from civil society, or the economic-centered perspective of “green growth”; also a technological fix for environmental issues that has prevailed in recent years; these ideas present growth and supply-centered pathways of no or marginal change to the status quo, respectively (e.g., Constance et al. 2018b; Friedrich et al. 2021a, 2021b, 2022; Longhurst and Chilvers 2019; Nightingale et al. 2020; Nowack and Hoffmann 2020; Soetebeer 2015). They are symbolic of the great importance of discourses and the role of dominant actors in shaping the foci and content of these discourses. In addition, this fact also shows that interviewees use different temporal framings when imagining the future (see also “Theoretical reflections and implications for the concept of imaginations”).

Nonetheless, what is visible in our case study, when considering the results of the modified nitrate directive and recent developments in relation to manure after use (Friedrich et al. 2021a), is that bioeconomic ideas are flourishing, thereby following the modernization trajectory (see also Friedrich et al. 2022). These ideas are strongly intertwined with the motivation of following the nitrate directive. This relationship indicates two things: first, the modification of regulations (in our case, the nitrate directive) can leverage new innovations to be developed and diffused, as argued by the interviewed innovation actors. Second, although different ideas of how to achieve sustainability (in terms of different “manure trajectories”) exist, these are not necessarily being negotiated in society, rather, economic actors are performing their imaginations in practice. In our view, this aspect may be related to power imbalances within the current system that may lead to the manifestation of existing (unsustainable) structures and the mental lock-ins of capitalist imaginaries in the livestock system, as we argue elsewhere (Friedrich et al. 2021a, 2022).

However, based on our empirical findings, we contend, with respect to other studies (e.g., Hochschild 2016; Neckel and Hasenfratz 2021), that the culture of societies and societal actors with respect to values and norms, emotions, and beliefs describes conflicting goals and imaginations and the associated practices and structures rather than factual knowledge. Our findings on different imagined sustainability trajectories also connect to existing political-economic ideologies of sustainability that are argued to inform decision-making on the individual and collective levels of societal organization (e.g., Davidson 2014). The transformation trajectory is informed by and linked to progressive ideas of social-ecological transformations that must apply to all sectors and dimensions of social life (e.g., Brand and Wissen 2018), while the modernization trajectory describes imagined technological fixes that will solve problems in the future and are often attributed to neoliberal ideas (e.g., Harvey 2003).

Our results must also be viewed in relation to the positionality of individuals and collectives in the spectrum of societal interests, as determined through their own economic interests and constraints. For our research, this aspect relates to the differing imaginations of NGO actors and innovation actors, as specified through their different trajectories. NGO actors do not have their own economic constraints in relation to developments in agrifood systems, whereas innovation actors may have invested in specific technologies, thereby developing innovations, resulting in the expectation that past economic investments must now be profitable (for further elaboration, see Friedrich et al. 2022). Thus, these past investments to some extent “colonize” the present (cf. Beckert 2018) of these actors and limit their ability to be interested in far-reaching changes, as such changes would jeopardize their business model. These investments and the necessity of future economic profitability can lead to path dependencies and lock-ins (see Friedrich et al. 2021a, 2022; Klitkou et al. 2015). In particular, farmers are often trapped by their past investments, and agriculture in general is heavily reliant on subventions (Barnes et al. 2016). All these aspects are major barriers to the design of sustainability-oriented transformations in general and with respect to German agrifood systems in particular, as they limit the ability to change.

In our view, different conceptualizations of sustainability are a double-edged sword. On the one hand, as long as these different conceptualizations of what sustainability means and how it can be achieved with social practices and sociomaterial structures exist in the debate on socioenvironmental issues, solving sustainability-related issues could remain difficult, as the differing trajectories, practices and structures are opposed or even antagonistic and could thus lead to concrete conflicts. Such conflicts would present additional barriers (to those discussed above) to the design of a sustainable livestock system. Examples of conflicts arising from contesting imaginations that embed conflicting social practices, which torpedo any conception of sustainability, can be found in various topics associated with sustainability transformations. One such example concerns the goal conflicts in the moderation of sustainable development goals (e.g., Schneider et al. 2019), exemplified by land-use conflicts such as those presented in the “food versus fuel” debate. Sociocultural conflicts between rural and urban regions are especially relevant to our topic, as the former regions are characterized by having the land that is required for change (WBGU 2020), while the latter are characterized by transformational imaginations about the future with respect to topics such as agriculture or energy transitions (e.g., Friedrich et al. 2021b; Gürtler and Herberg 2021; Nowack and Hoffmann 2020). These differences can escalate toward what Gürtler and Herber (2021) call “moral rifts” that rest in diverging perceptions of justice and that may lead to resistance to change, which again torpedoes any imaginations of sustainability. Against this background, it is thus important to uncover conflicting imaginations to foster societal exchange and discourse that builds the foundation for the co-design of approaches and strategies that solve socioenvironmental issues and moderate different conceptions of sustainability.

On the other hand, it is the very nature of transformation processes that they are leveraged and accompanied by conflicts (e.g., Skrimizea et al. 2020)—or, as we argue above, that these conflicts could present a process of contingency building (cf. Reckwitz 2021). In addition, theories from sustainability transition literature (such as the different transition pathways of Geels and Schot (2007), or the cultural evolution of a sustainable bioeconomy, (e.g., Schlaile et al. 2021) show that different innovations (which can be part of different trajectories) can also complement each other and cumulatively shift existing regimes. This possibility would mean that the different trajectories are a symptom of an ongoing transformation rather than necessarily resulting in concrete (escalatory) conflicts. To leverage sustainability transformations against the background of ongoing global environmental change, these questions must receive further attention from the scholarly community.

### Methodological reflections

We have outlined that we did not detect a control trajectory and attributed this lack of detection to the specific case of agrienvironmental discourses. However, other reasons are possible as well. Our sampling strategy, which relied on snowball sampling, could have led to a bias in that governmental and state actors (which Adloff and Neckel (2019) designate as representatives of the control trajectory) are excluded from the sample. Other biases could also originate from the sampling strategy, as we built upon problem-centered interviews. Therefore, actors, for example, those who design control practices, which are not necessarily argumentatively tied to the problem of manure surplus, are excluded from our sample. We encourage scholars to conduct further research on sustainability trajectories in the livestock system that quantitatively tests our identified trajectories and discerns whether they point to subsequent conflicts or are drivers of or barriers to change. In addition, future research could further explore conflicting practices as examples of “doing contingency” (Reckwitz 2021) and apply Adloff and Neckel’s (2019) framework to other socioenvironmental contexts to further substantiate the empirics of the theory.

## Conclusions

The aim of this study was to identify different trajectories of manure futures using a case study, thereby empirically proving the applicability of Adloff and Neckel’s (2019) conceptual framework and drawing conclusions regarding the development of a sustainable livestock system. Our results show three different trajectories that include opposing imaginations of manure futures: preservation, modernization, and transformation. Thus, our study proves that the conceptual framework of Adloff and Neckel (ibid.) is applicable to specific environmental topics, thereby allowing us to bridge the gap between future imaginations, current social practices and their mutually dependent sociomaterial structures. The empirical nature of our case study enabled us to extend the framework to grasp the construction of specific imaginations. In this respect, we identified the meanings attributed to actors as determining factors for the different trajectories. This category is underrepresented in the original conceptual framework and must therefore be considered in further research. In addition, our results show that the trajectories present ideal types of change that do not exist in reality, rather, the actors show hybrid versions of the identified trajectories.

In terms of practical implications, we found that the different trajectories, including their opposing imaginations, practices and structures, could present a barrier to the design of a sustainable livestock system. As long as differences in imaginations lead to differences in practices and structures, such differences can lead to conflicts. In general, it is the very nature of transformation processes to be accompanied by conflicts. Whether these conflicts are drivers of or barriers to change remains an open question. In our view, it is important to moderate these conflicts as drivers of change. As we have shown, research on imagined futures can enrich this question by answering it in terms of disclosing the content of imaginations of subjects and collectives and how these imaginations shape social practices and mutually dependent sociomaterial structures.

## Footnotes

1 We understand both imaginations and imaginaries as being socially embedded and thus use the terms synonymously in the following; see also Adloff and Neckel (2019).

2 The concept of sociotechnical imaginaries was initially developed by Jasanoff and Kim (2009) to analyze “collectively imagined forms of social life and social order reflected in the design and fulfilment of nation-specific scientific and/or technological projects”. This definition was further adjusted by Jasanoff (2015) to accommodate the visions of different groups of stakeholders or collectives in aiming to create a desirable future.

3 Adloff and Neckel (2019) do not explicitly exclude this view, as imaginations are embodied in social practices, which are foremost reproducing orders of knowledge and cultural interpretive patterns (cf. Reckwitz 2021, p. 56f.). However, Adloff and Neckel (2019) do not clearly highlight that they understand imaginations as socially constructed (fragmented) reality through a social constructivist approach, whereas we are highlighting this point in the following, thereby following the call of Longo et al. (2021) to include social constructivist and social realist approaches in sustainability science.

4 We focus on the shared understandings of meanings and knowledge rather than individual differences that may be created through the situatedness and positionality of individual subjects.

5 We refer to the sociotechnical regime as the current existing dynamic-stable coalescence and interplay of different actors such as consumers, science, and economy of a particular sector or topical issue. This view highlights their (embodied) rules and (social) practices (for more details, see Geels and Schot (2007)).

6 As the interviews were conducted in German, the excerpts used in this article have been translated to English by the authors.

7 These path dependencies exist on different levels of societal organization and are complemented by international and global developments, such as those described through the concept of food regimes; see McMichael (2009).

8 Orders are being produced through “contingency closure processes” (“doing consistency”) that challenge existing orders (“undoing orders”) in how social practices are arranged and embodied by humans. Examples of orders include “neoliberalism” or “socialisms” (Reckwitz 2021, p. 72ff.).

9 Social practices, in this respect, include both sociotechnical and economic practices.
